# Pharmaceutical Aspects of Artificial Nutrition

**DOI:** 10.3390/jcm8112017

**Published:** 2019-11-19

**Authors:** Emilie Reber, Markus Messerli, Zeno Stanga, Stefan Mühlebach

**Affiliations:** 1 Department for Diabetes, Endocrinology, Nutritional Medicine and Metabolism, Bern University Hospital and University of Bern, 3010 Bern, Switzerland; zeno.stanga@insel.ch; 2 Department of Pharmaceutical Sciences, Pharmaceutical Care Research Group, University of Basel, 4050 Basel, Switzerland; markus.messerli@unibas.ch; 3 Department of Pharmaceutical Sciences, Division of Clinical Pharmacy & Epidemiology/Hospital Pharmacy, University of Basel, 4050 Basel, Switzerland; stefan.muehlebach@viforpharma.com

**Keywords:** parenteral nutrition, enteral nutrition, artificial nutrition, all-in-one parenteral admixture, compatibility, stability, pharmaceutical expertise, drug admixing, drug administration

## Abstract

Artificial nutrition, including enteral (EN) and parenteral (PN) nutrition, is indicated whenever adequate oral nutrition fails to sufficiently supply the necessary nutrients to the body. It is a convenient, efficacious, safe, and well-tolerated form of clinical nutrition in the hospital and home setting. EN is administered via nasogastric tube or ostomies while PN usually requires a central venous access for administration, straight into the blood stream. The infused nutrients can then be taken up directly by the different organs. PN is targeted as a single daily portion formulated as an oil-in-water emulsion providing the necessary substrates for the catabolic and anabolic metabolism including macro- and micronutrients and fluids. PN has a complex pharmaceutical composition—all-in-one admixture—and its compounding or ready-to-use preparation. The use of PN is more challenging and more expensive compare to the use of EN, commercially available as ready-to-use formulations. EN and concomitant medication is highly challenging. Upon incorrect handling and administration, PN is associated with potentially severe or even fatal complications, mostly relating to the central venous access (e.g., catheter-related sepsis) or to a metabolic intolerance (e.g., hyperglycemia, refeeding syndrome) because of inappropriate administration. A correct order of admixing, correct dosing, and administration of the artificial is crucial for safety and efficacy; clinical and biochemical monitoring of the patient and treatment regimen adaption are necessary. The high number of reactive solutes allow only limited stability of a ready-to-use PN admixture. The potential for numerous incompatibilities and interactions renders PN admixtures generally unsuitable as drug vehicle. Laboratory compatibility and stability testing and pharmaceutical expertise are a prerequisite to define the PN composition including nutrients or even drugs admixed to define the appropriate and individualized nutrition and medication regimen. The aim of this narrative review is to present the actual state-of-the-art to deliver best quality artificial nutrition with special regard on pharmaceutical aspects such as instabilities, incompatibilities, and concomitant co-medication.

## 1. Introduction

Enteral nutrition (EN) is used whenever the gastrointestinal tract is functioning and when oral access is impaired (e.g., chewing and/or swallowing issues). In patients with partial or total intestinal failure, nutrients may not or not be sufficiently absorbed from the intestine. Hence, parenteral nutrition (PN) has to be administered as a formulation, containing the necessary substrates, ready to be used in the intermediate metabolism. Intestinal failure may result from an extensive surgical bowel resection or a disease leading to reduced function of the intestine and/or impairment of motility, digestive or absorptive capacity (e.g., mesenteric infarction, laparoschisis, Morbus Hirschsprung). Table 1 shows recently published data from Pironi et al. regarding epidemiology of intestinal failure in 3239 patients across the world [1]. PN allows quantity- and quality-wise a full or partial nutritional support and can guarantee survival and good quality of life. EN and PN are the two forms of artificial nutrition, developed and introduced in the 1960s. EN and PN, or their combination, require an adapted and individualized nutrition regimen respecting the specific condition and requirements of the patient. Nowadays and in contrast to the early beginnings, EN and PN are mostly based on physico–chemically fully defined, balanced and stable products, manufactured in industry. While EN products can be delivered as stable ready-to-use formulations, total or partial PN has to be compounded or prepared ready-to-use for administration compliant with pharmaceutical good manufacturing practice (GMP) requirements. The available industrial PN premixes contain stable nutritional components mechanically separated from each other in chambers with breakable sealing. Upon ready-to-use preparation as all-in-one (AiO) admixture in a convenient, single container and for single line daily PN treatment, the sealing is mechanically broken and the content manually shaken. For safety and tolerance of administration of the usually hypertonic PN admixtures showing an osmolality exceeding 2000 mosm/kg, an inserted/implanted central intravenous access is required [2]. Consequently, PN and its prerequisites are more challenging, more expensive, and more prone for complications compared to EN. Nevertheless, a well-indicated PN according to existing guidelines shows good efficacy and safety in patients, also in long lasting home PN and if assisted by a nutrition support team (NST) [3,4,5,6,7,8].

Databases such as PubMed and Cochrane were searched for guidelines, recommendations and registries, using filters for human studies in English only, and excluding children, as well as homepages from national and international nursing and nutritional societies. The aim of this narrative review is to present the actual state-of-the-art for artificial nutrition delivery with special regard on pharmaceutical aspects. This review aims to provide nutritionists dealing with artificial nutrition knowledge to deliver best quality PN, understanding of the concept and benefits of the AiO, and the pharmaceutical challenges of artificial nutrition (instabilities and incompatibilities, drug admixture).

## 2. Accesses for Artificial Nutrition

PN should be initiated at the latest after five to seven days of insufficient oral and/or enteral feeding. This may be initiated even earlier in case of severe malnutrition [5]. Preoperative PN and also EN have been for example been shown to improve surgical outcome in patients affected by Crohn’s disease undergoing abdominal surgery [9,10]. The beneficial value of supplemental PN in critically ill patients has also been demonstrated in recent prospective studies [11]. EN may be administered via nasogastric tubes, nasojejunal tubes or percutaneous gastrostomy/jejunostomy (endoscopic or radiologic), Witzel fistulas or fine needle catheter jejunostomy, depending on the anticipated duration of the EN therapy and of the indication. The need for a central venous access for PN is obvious when for example calculating the individual daily requirements for electrolytes, which have to be contained in an AiO PN admixture. These alone increase the tonicity by more than 600–800 mosmol/kg, which is the maximal value for a peripherally administered intravenous infusion [5]. In long-term PN, tunneled subclavian or jugular catheters (e.g., Hickman catheters), implanted port systems or peripherally inserted central venous catheter (PICC) are used [6]. For short period of supplemental PN, the administration of peripheral PN admixtures through peripheral venous catheter is possible. Peripheral PN may be indicated for short-term use or as a supplement typically to maintain a previously well-nourished, patient or to serve as a bridge to centrally administered infusions or until adequate enteral feedings can be established [12,13]. The risk of microbial contamination and following growth is greater with peripheral PN than with PN, mainly due to the lower osmolarity in peripheral PN [14]. Moreover, the risk of phlebitis and extravasation is high and causes catheter removal [15].

## 3. Handling of Feeding Tubes and Catheters

The handling of feeding tubes is much less demanding than the catheter handling, as it does not require asepsis. It however largely depends on the type of tube. Regular dressing changes and slights moves of the tubes are however mandatory.

To minimize PN-associated complications, the appropriate central intravenous device has to be selected, placed, and inserted by a trained surgical team with experience. Together with good and regularly trained catheter handling, these are the two most important factors to keep the main intravenous access related complications in PN low. A rigorous aseptic technique is required for the manipulation and care of the catheters and the connections [2]. Blood drawing including aspiration through the central venous catheter has to be avoided. Suited trainings for patients and caregivers to learn the defined rules and best practices are essential and must be documented and evaluated [16]. Such education includes aseptic handling of the PN bag, of catheters, and its connections including also a rigorous handwashing procedure. Alcoholic chlorhexidine (0.5–2%) is recommended for skin disinfection; there are possible disinfectant alternatives in case of contraindications [2]. When a port system is used for PN, the giving set must be changed daily and in addition, the gripper needle has to be changed every three to seven days according to accepted standards. Evidence-based policies also apply for dressings of the exit sites [2]. The rinsing and plugging of the catheter with defined solutions are of critical importance and saline (0.9%) is the standard solution to be used. Heparin, initially recommended for port system rinsing, is still often used despite lacking appropriate compatibility documentation. Bozetti et al. showed a significant increase of catheter-related complication using heparin rinse compared to saline in HPN patients [17]. A recent Cochrane update analysis comparing saline against heparin for intermittent catheter locking in adults showed no evidence for a heparin benefit, as known from previous studies in children [18]. Heparin is prone to incompatibility reactions with many PN components, e.g., lipids, potentially leading to occlusion and/or infection of catheters. The use of heparin to rinse catheters lacks evidence regarding its effectiveness in reducing blood clotting. Moreover, it is prone to incompatibilities with lipids and emulsifiers from the PN admixtures. Even short and at low dose, heparin exposure in an intravenous line has shown emulsion cracking [19]. As a consequence of the cracking, lipid deposits form in catheters increasing risk for infectious complication and obstruction, possibly leading to catheter removal [6]. The potential risk to induce heparin hypersensitivity has also to be considered. In the actual PN guidelines, heparin is not more recommended for catheter rinsing and plugging [2,6,18]. A lock solution with taurolidine, a synthetic antimicrobial agent, might be considered in PN patients with repeated catheter-associated infections but should not be routinely used [20]. Other antibiotic lock solutions (vancomycin, gentamycin) are also used although the evidence for a preventing effect is low. Ethanol locks may be considered for secondary prevention in some cases. The use of in-line filters is not supported by the necessary evidence, similarly to heparin use or antibiotic prophylaxis [2]. Therefore, these methods are not considered as universal and compulsory preventive measures for all PN patients [5,6,7]. In-line filters may be used to (1) filter out precipitates or particles (reduce risk of embolism), (2) prevent pathogenic microorganisms to enter the bloodstream (reduce risk of infection), (3) hinder air to be infused into blood circulation (air embolization). In-line filters may however also create new problems [21]. They may themselves release particles or cause adsorption of nutrients or drugs reducing their systemic availability. In-line filters may thus also potentially be blocked and impair the PN administration. A fatality has even been associated with the use of in-line filters, as calcium phosphate precipitation occurred in the filtered admixture after warming up in the line [22]. There are no general recommendations for the use of in-line filters. German guidelines on PN settle beneficial effects of in-line filters in specific risk patient populations (e.g., children and immune-compromised patients) [23]. The ASPEN however recommends the use of 0.22 micron filter for lipid-free PN admixtures, and 1.2 micron filter for AiO admixtures [24]. The filter should be placed as close to the patient as possible and should be exchanged with each new PN container, as well as the administration set.

## 4. Complications of Enteral Nutrition

EN is generally efficient, safe and well tolerated. Small bore tubes made of more flexible materials, careful nursing and therapy monitoring contribute to decrease the complication rates. Risk factors for complications are among others neurological impairment, anatomical abnormalities, and advanced age [25]. Systematic antibiotic therapy before tube placement to prevent infectious complications (wounds and systemic infect) is controversially discussed. There is however good evidence for at-risk patients (e.g., immunocompromised patients) and placement of gastroscopic accesses [25]. Administration of a single dose of broad spectrum antibiotic half an hour before EN device placement has been shown to reduce peristomal infects by 22%. Complications rates vary according to the type of access, e.g., 0.3–15% for nasoenteric tubes, 1–4% for major and 13–40% for minor complications for endoscopic accesses. Complications rates are very low for surgical needle catheter jejeunostomies.

### 4.1. Gastrointestinal Complications

Gastrointestinal complications are frequent with enteral feeding [26]. Diarrhea may be the most common one, probably caused by adaption of the intestine after fasting period, by antibiotic therapy, or administration of too cold solutions. Delivery sites and rates influence may also influence the occurrence of diarrhea. Misplacement (too low) may be a further cause of diarrhea. Obstipation may also occur with enteral feeding solutions containing low or no dietary fibers, due to dehydration, immobility, or as a side effect of opiate therapy.

Nausea and vomiting may be due to tube dislocation, too fast administration or administration of too high volume. It may also be caused by the underlying disease or the related treatments, or due to medication containing sugar substitutes. Delayed gastric emptying and subsequent feeling of fullness are risk factor for aspiration and may also cause vomiting [25].

Reflux esophagitis and early dumping are further complications of EN.

### 4.2. Mechanical Complications

Feeding tubes may be misplaced, dislocate, or cause perforation (trachea or gastrointestinal tract), and nasal/gastrointestinal bleeding. Further, feeding tubes may be obstructed. Obstruction may be prevented by sufficient and consequent rinsing procedures, and by avoiding drug administration (as far as possible). There are various possibilities to re-open a tube, from warm water rinsing to rinsing with pancreatic enzymes and bicarbonate solutions. Feeding tubes may also cause irritation, and consecutive changes in the oral mucosa. Leaks or buried bumper syndrome may occur with PEG devices [25].

Aspiration is a rare complication (1–4%), causing fever and possibly pneumonia [25]. It may be silent or manifest with symptoms such as tachypnoea, tachycardia, and wheezing. Aspiration is however a great issue in critically ill patients [26]. It may be prevented elevating the head by 30–45° when lying [25].

### 4.3. Infectious Complications

Different infectious complications may occur during the EN therapy, such as pneumonia (arising from aspiration pneumonia), sinusitis, or bacterial contamination of the nutritional solution [26]. Some complications are related to the device, e.g., wound infection at the entry site with PEG devices and peritonitis with gastroenterostomies. Infections mostly require tube change/removal [25].

### 4.4. Metabolic Complication

The metabolic complications of the enteral nutrition are similar to the one of parenteral nutrition (e.g., the refeeding syndrome described in Section 5.3.1) but with much lower incidence and severity [25].

## 5. Complications of Parenteral Nutrition

### 5.1. Mechanical Complications

Catheters may sometimes dislocate or occlude, mainly due to incorrect manipulations. Reasons for catheter occlusions are manifold: PN admixture instabilities, incompatibilities with rinsing solutions and/or with other intravenous solutes administered through the catheter, etc. Taking blood samples through the central venous access must be prohibited since it is almost impossible to completely clear the line and the device afterwards. Moreover, blood samples from catheters often provide erroneous results because of residues of the PN in the catheters with high electrolyte or lipid concentrations. Blood clots may form and eventually mechanically occlude the catheter, being a possible origin of microbial colonization. Occluded catheters may be rinsed with saline solutions (10–20 mL) in a first step, applying slight pressure. If this is not successful, acidic or alkaline solutions may be applied according to defined procedures [2]. Reopening of occluded catheters is however discussed very controversially by the experts. It can be dangerous since clots may be microbially colonized. Blood stream infection may consecutively occur. Lipid deposits may be eliminated with ethanol or diluted sodium hydroxide. Thrombolytics are administered to eliminate an assumed blood clot [5,6]. Administration of PN with infusion pumps also helps to prevent catheter occlusions since the flow rate is kept constant [7].

### 5.2. Infectious Complications

In the beginning of PN history, infections and sepsis were frequent (affecting up to 40% of the patients) and thus a limitation to PN use [8]. By reducing the number of manipulations (e.g., AiO admixture administration), the incidence of catheter sepsis radically dropped (Table 1). Catheter-related sepsis occur at rates of 0.5–1 per catheter year in hospitals inpatients and 0.1–0.5 per catheter year in patients on home PN [5]. Gram-positive microbes (from the skin) are the mostly encountered microorganisms identified [2,27]. Catheter exit site-related or bloodstream infections are predominant complications and are associated with increased morbidity and mortality rates. Moreover, infectious complications contribute substantially to PN costs, causing additional hospitalizations and catheter removals.

### 5.3. Metabolic Complications

#### 5.3.1. Refeeding Syndrome

The refeeding syndrome is a potentially life-threatening metabolic condition occurring in seriously malnourished patients or in patients recovering from severe catabolic diseases (e.g., sepsis, diabetic ketoacidosis) after start of a nutritional therapy. From a pathophysiological point of view, refeeding syndrome is an exaggerated response of the malnourished catabolic body to a nutritional therapy, indeed to anabolism [28,29]. Refeeding syndrome is characterized by severe electrolyte shifts (mainly hypophosphatemia, hypomagnesemia, and hypokalemia), vitamin deficiency (mainly thiamine), fluid overload and salt retention leading to organ dysfunction including cardiac arrhythmias up to death. Symptoms such as heart failure, peripheral edema and neurologic disorders can occur. The refeeding syndrome is most likely to appear within the first 72 h after initiation of the nutritional therapy. A risk stratification of patients before prior to start a nutritional intervention is recommended and can then by adapted according to the risk category [28,29]. Full energy requirements are targeted within five to ten days after initiation of the nutritional therapy depending on the risk category, starting at a low energy rate (5–15 kcal/kg/day) and increasing stepwise. Fluid management and sodium restriction may be necessary in patients belonging to higher risk categories. Thiamine (vitamin B1) has to be administrated as a 200–300 mg dose daily for three days, 30 min before the initiation of nutritional support. Finally, provision of trace elements and vitamins in the single and double recommended daily amounts of micronutrients, respectively, are recommended. Intensive clinical monitoring is mandatory to detect early signs of refeeding syndrome, such as organ failure and fluid overload. It should include vital signs, hydration status, and determinations of serum electrolyte levels.

#### 5.3.2. Hyperglycemia

Hyperglycemia is another early and frequent complication affecting up to 50% of the patients upon PN initiation. Appropriate and initially frequent glycaemia testing is mandatory, since hyperglycemia has been linked with increased morbidity and mortality, especially in critically ill patients [30]. The targeted blood glucose level lies between 7.8 and 10.0 mM, (normoglycemia: 4.4–8.1 mM; 80–145 mg/100 mL) [31]. Insulin administration, optimally pump-assisted and in parallel to PN, may be necessary to control glycaemia (there is a dedicated specific article on this topic in this special issue [30]).

#### 5.3.3. Liver-Associated Complications

Liver-associated PN complications are seen in up to 40% of the patients, especially in those with short bowl with less than 150 cm of remaining small intestine and in absence of colon (Table 2) [2]. In case of occurring liver-associated PN complications (e.g., hypertriglyceridemia, cholelithiasis, and cholestasis), it is recommended to administer PN formulations with reduced triglyceride (TG) content and a better fatty acids (FA) mix (e.g., by a higher monounsaturated FA (MUFA) content or an increased Ω-3 to Ω-6 ratio of the polyunsaturated FA (PUFA)) [2].

#### 5.3.4. Thrombosis

Central venous thrombosis is a common issue in PN patients with central venous access (up to 50% of PN patients) associated with high morbidity and mortality rates (Table 2). The localization is mostly proximal to the catheter. The frequency of thrombosis is linked to the experience and skills of the catheter insertion team as well as to the diameter of the catheter; small bore central venous catheters are therefore recommended. Low dose oral anticoagulants may be used in high-risk patients [6].

## 6. The Role of the Pharmacist and Specificities of Pharmaceutical Management

NSTs have multi-professional composition consisting of at least a physician, a specialized nurse, a dietician and a pharmacist, skilled to manage PN, and to deliver best nutritional support [16]. Pharmacists within the NST have an important role to play in selecting, preparing and instructing on the safe handling of nutritional products, especially PN. They have however also a clear role in optimizing medication for patients with EN (BN Group). Pharmacists provide medicinal products, care, and when necessary education and training related to artificial nutrition to the other NST members, to patients and their caregivers. Pharmacists are in charge for the logistic of the products and of their quality assurance. They check the drug and nutrients prescriptions from a pharmaceutical point of view, advising on the most effective and safe administration of drugs in order to prevent interactions and incompatibilities, in EN (administration via feeding tube) as well as in PN (admixture) [32]. Pharmacists are also in charge of the documentation and clarification of drug related adverse events, to increase treatment safety. Most patients on artificial nutrition also require drug treatment for their underlying diseases. This further complicates the overall treatment regimen aiming in given cases to combine the parenteral administration of nutrients and drugs, e.g., as the indication for (home) PN is extended to malignant chronic diseases or severe functional deficiency, often requiring additional complex medication in parallel to the artificial nutrition. This is a complicated endeavor, which needs a careful check of compatibility primarily respecting correct and suited dosing over time of both nutrition and medication to ensure safe and efficacious treatment requesting pharmaceutical skills [6,8,33]. Drug admixing issues are one of the main tasks of the pharmacist within the NST, who has to face and assess PN- and drug-related problems from the pharmaceutical perspective [32]. The pharmacist also contributes to define an appropriate nutritional and medical care plan, to avoid medication errors, and finally keeps responsibility that the right patient gets the right products administered in the right way [5,6,34].

## 7. Components of Artificial Nutrition

Standard commercial nutritional solutions for enteral use contain between 1 (isocaloric) and 2 kcal/mL (hypercaloric), with 15–20% proteins, 25–30% fats, and 50–60% carbohydrate, which represents a suitable macronutrients distribution for most patients. They may contain dietary fibers or not. Macromolecular and low-molecular weight solutions are available depending on the functionality of the gastrointestinal tract. Additionally, metabolically adapted solutions (e.g., high protein, low electrolyte content for kidney failure patients) or immunomodulating solutions (containing e.g., arginine or glutamine) are available. Organic amino acids (AA; protein), glucose (carbohydrate), different TG of FA (fat), and inorganic/organic electrolytes/nutrients together with water are the small molecular components of a PN [2]. Micronutrients, vitamins and trace elements have mostly to be added for a total PN as they are not necessarily included in industrial multi-chamber bags. Energy requirement in adults is 25–30 kcal/kg/day given as a mix of the most important universal fuel glucose and high caloric lipids. In contrast to EN, the AA content of PN is not calculated as energy as AA are primarily intended building components for protein synthesis which has to be considered when assessing the balance of EN and PN. The basic protein need in adults is 0.8–1 g/kg/day increasing to 1.2–1.5 g/kg/day in malnourished patients and even higher in special situations (e.g., 2.5 g/kg/day in burned patients or children). The energy need mainly depends the resting energy expenditure and on disease activity and severity (possibly increasing the requirements by 50%).

### 7.1. Amino Acids

In a severe catabolic state, glucose may be produced from AA over the formation of acetyl-CoA and through the Krebs cycle or over the pyruvate gluconeogenesis pathway (for glucose-dependent organs, e.g., brain), yielding in 1 g AA = 4 kcal. The protein (AA) breakdown may be estimated through the urine output, since nitrogen from the AA is eliminated as urea. One gram of urea contained in the urine matches 7.34 g of AA (0.47 g nitrogen). This calculation can be used for an intake-output estimate e.g., in well-monitored critically ill patients.

From the 21 AA, there are seven essential (isoleucine, leucine, lysine, methionine, phenylalanine, threonine, tryptophan, and valine) and four conditionally essential AA (histidine, tyrosine, cytosine, glutamine, and taurine). When AA show additional pharmacological effects, the term pharmaconutrition is used. This applies to glutamine, although the evidence for effectiveness is still debated. Commercial AA solutions for PN are traditionally crystalline solutions of L-AA (10%), despite differences between the manufacturers in the conditionally essential AA content (mainly glycine) [2]. AA in solutions are filled in airtight containers and protected by the antioxidant nitrogen gas, since oxygen oxidation (ambient air) is very likely to occur. AA and glucose put together in vitro can react and undergo Maillard reaction, which may influence their availability. Thus, a yellow to brownish colored product is then visible upon exposition to ambient conditions as e.g., beyond 24–48 h hanging time of ready-to-use AiO admixtures. The AA content in a daily dose of AiO PN admixture reaches 1.2–1.5 g/kg (100–150 g AA). In order to reverse catabolism (AA breakdown), AA should be administered together with fat/carbohydrates (mainly glucose). The suggested caloric intake is 20–27 kcal/g AA [5,7]. Optional admixtures of pharmaconutrients (e.g., 0.2 g glutamine/kg/day in trauma or burned patients) are dosed additionally to the necessary AA amount of the PN regimen and have to be taken into account in the nutrient balance.

### 7.2. Glucose

Around 66% of the body’s energy fuel is normally provided by carbohydrates (1 g = 4 kcal), mainly by glucose, the primary and physiological energy substrate in the intermediate metabolism. It is prone to oxidative degradation when in solution. Some organs (e.g., blood and brain) fully depend on glucose for meet their energy requirements. Hence, mechanism such as gluconeogenesis ensures a minimal necessary glucose production (37 g/day) when external supply is insufficient or lacking and the restricted glycogen stores (150–300 g) empty [2]. Highly concentrated, hypertonic glucose solution is used to restrict the volume of an AiO PN admixtures and primarily contributing to their hyperosmolarity. Maximal infusion rate in adults is 3–6 g glucose/kg over 24 h. This rate is limiting the maximal infusion rate since the rate of glucose oxidation to pyruvate/acetyl-CoA/Krebs cycle in adults is limited to 5–7 mg/kg/min [2]. The level of blood glucose during PN has to be kept <10 mM and has to be regularly monitored, especially in the beginning of PN [31,35].

### 7.3. Lipids

Commercial intravenous lipid oil-in-water emulsions contain 100–200 g lipid/L [2]. The TG used are composed of different (PU)FA. The oil-in-water emulsion is stabilized with soya or egg yolk lecithin (12 g/L), a phosphatidylcholine with a negative surface charge resulting from the phosphate groups [36]. The surface charge is negative from the anionic phosphate moiety of the emulsifier at the surface of the oil droplets. This negative zeta potential keeps the emulsified oil droplets separated. The anionic charge of the phosphate lecithin moiety at the surface is critical for destabilizing incompatibilities, e.g., with (mainly polyvalent) cations. Intravenous lipid emulsions are nearly isotonic and contribute to decrease the osmolarity of an AiO PN admixture, e.g., in PN administered peripherally. Important to know, about 15 mmol phosphate are delivered from the emulsifier per liter of a commercial parenteral lipid emulsion [2]. TG are important energy fuels providing 9 kcal/g through the beta-oxidation of FA and the subsequent acetyl-CoA metabolism. Different FA types are contained in intravenous lipid emulsions, depending on the lipid sources. Nowadays, “structured lipids” are mostly being used, like SMOF lipids containing 30% soybean FA, 30% MCT, 25% olive oil FA, and 15% fish oil FA. Lipid emulsions dose in PN reaches 0.5–1.0 g/kg/day to cover about 33% of the patients’ energy requirements. Lipids may be used to a higher extend in critically ill patients (up to 50%) to avoid insulin resistance issues [2]. Since they are partially essential FA, PUFAs must be included in AiO PN admixtures in the required doses. Ω-3 PUFAs like EPA (eicosatetraenoic acid) or DHA (docosahexaenoic acid) show anti-inflammatory effects through the synthesis of prostaglandins and leukotrienes and may be considered as pharmaconutrients. On the contrary, Ω-6 PUFAs shows pro-inflammatory effects forming arachidonic acid over the prostaglandins and leukotrienes pathways. Ω-9 MUFA have no action on these pathways and thus are neutral. Middle chain triglycerides (MCT) only contain non-essential saturated FA. They can be oxidized to produce energy directly in the mitochondria in absence of carnitine unlike long chain PUFAs. PUFAs are highly prone to peroxidation; resulting in toxic reaction products (e.g., radicals and aldehydes) contributing to the systemic inflammation and the oxidative stress of patients. Light and oxygen protection for storage and transport are therefore important to prevent peroxidation [37,38]. The oil droplet characteristics and distribution in the oil-in-water emulsion is a critical parameter. It should mimic the physiologic chylomicrons or lipoproteins with a critical upper diameter size of 5 μm correlating to a small blood vessel diameter. Larger droplets may eventually cause lipid embolism, while degradation products of lipid peroxidation may as radicals free cause DNA damages and contribute to inflammation [2,16].

### 7.4. Fluids and Electrolytes

Sufficient water and electrolytes doses have also to be provided by a nutrition regimen. Additional oral fluid or infusions to PN may be needed to reach the basic fluid requirements in adults of 30–40 mL/kg/day, but also to cover abnormal losses such as fever, vomiting, diarrhea, or stoma losses, burns and severe wounds [39]. The combination compatible amounts of (di- and trivalent) cations and anions in AiO admixture preventing instabilities e.g., by harmful precipitations of salts or deteriorated oil-in-water characteristics by interactions with the emulsifier is a pharmaceutical challenge.

### 7.5. Micronutrients

Daily administration of EN > 1500 mL covers the daily recommended micronutrients intake. Since most micronutrients are hydrophilic and body stores are limited, total PN must also cover vitamins and trace elements requirements from its initiation. Trace elements (polyvalent cations) show relevant and concentration-dependent physicochemical interactions e.g., with the oil-in-water emulsifier; they may also be catalyzers of chemical degradation processes (e.g., oxidation) [2]. This may become an issue since vitamins and trace elements are both infused into AiO PN bags. As an example, iron or copper catalyze the oxidation of the ascorbic acid (vitamin C), which then degrades within minutes. From an evidence-based standpoint, trace elements and vitamins should be administrated separately, e.g., trace elements admixed to the AA portion, and vitamins only given at the end of a PN administration limiting the exposure time of combined physical presence in the admixture [4,5,6].

### 7.6. The All-In-One Concept as the Pharmaceutical Formulation of Choice

PN is a complex, meta-stable, high quality pharmaceutical formulation defined by the pharmacopoeia. In presence of lipid, it represents an oil-in-water emulsion and contains various, partially ionized, reactive solutes, prone for physicochemical interactions. PN is highly concentrated (hypertonic) and its components are often close to their solubility limits as the volume is restricted [32]. A PN regimen has to be practicable and convenient, efficacious and safe, also upon long term use (home PN). Central venous access is needed for the administration and aseptic techniques are needed during ready-to-use preparation of industrial premixes or tailor-made compounding since PN has to be sterile and pyrogen-free [6]. Correct conditions for transport and storage and finally for the administration and hanging time are required to provide the right and ready to be metabolized amount of nutrients to fulfil the nutritional needs and to prevent and/or correct metabolic/physicochemical disturbances.

The PN composition and if needed its individualization has always been and is a challenge to ensure safety and optimal efficiency. The increased knowledge of disease and stress metabolism has contributed to diminish complications and to increase tolerance of PN [2]. Historically, PN started from the difficult to handle multi bottle to a convenient single container AiO system delivered as a ready-to-use complete daily portion enabling individualized, more physiologic and well-tolerated 24 h or cyclic co-administration of the nutrients (mostly overnight) [4,40].

**Figure 1:**
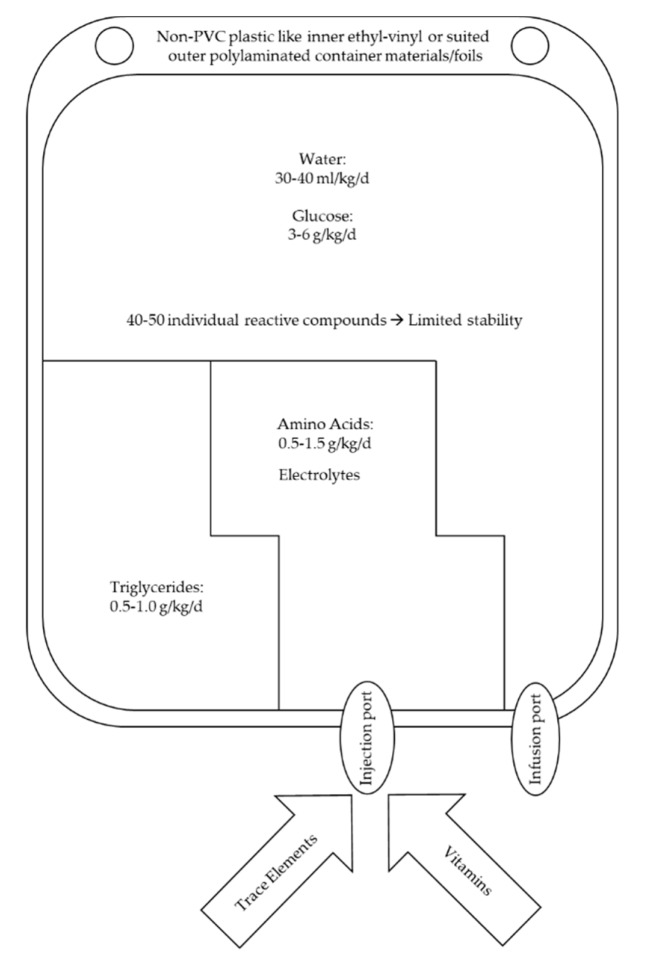
The all-in-one PN concept with adult nutrient requirement, adapted from [2].

Total PN admixtures and regimens contain over 50 individual solutes, mostly representing reactive species (Figure 1). This explains the stability issues and the important potential for physicochemical interactions such as degradation of components, generation of toxic products like reactive aldehydes from fatty acid degradation or harming precipitates formed from interacting electrolytes [2]. Additionally, a critically reduced homogeneity of the oil-in-water fat emulsion with eventual oiling out may cause serious adverse effects. The control and avoidance of microbial contamination are key issues to reduce infectious complications. Nowadays, pharmaceutical GMP rules are established and require for example to work in a laminar airflow bench for aseptic compounding/ready-to-use preparation. There are guidelines for correct labelling, storage, hanging time (24 h after ready to use preparation), transport and storage (2–8 °C) of AiO PN admixtures [4]. The combined admixing of potentially interacting or even incompatible microelements like trace elements with vitamins are still debated. Trace elements can catalyze oxidation and radical formation of e.g., vitamins [4,6] or of PUFA [37]. An acceptable, appropriately documented compromise has to be defined for the appropriate quality of the AiO admixture administered and the provision of the necessary amount of nutrients. Individualized compounding or ready-to-use preparation of a complete PN results in an AiO admixture, which cannot to be sterilized anymore by an established heating procedure [2]. Hence, in many countries, ready-to-use AiO PN has to be prepared according to the GMP rules and regulatory authorization is needed. There are however countries where extemporaneous parenteral nutrition preparations do not need any specific regulatory authorizations. The industrial approach with serial manufacturing targeted stable and storable PN products to overcome the stability issues of individually compounded AiO PN admixtures. This resulted in the development of different forms of special multi-chamber bags, separating the lipid emulsion, the AA and the glucose solutions from each other by mechanically separated chambers or compartments [2]. The bags materials are innovative multi-layered foils allowing vapor sterilization of the filled and sealed chambers representing AiO PN premixes which are with the additional air-tight wrapping including oxygen absorbing materials stable up to years as the main chemical destabilization by oxidative degradation of the nutrients is almost eliminated [40]. Ready-to-use preparation of these commercial AiO PN premixes includes mechanical breaking of the chamber sealing and manual shaking of the combined content still in a closed container envelop thus in aseptic conditions. Admixing of other needed components (electrolytes, micronutrients, or other intravenous supplements according to need and compatibility) into individual chambers require suited stability data, and defined and validated admixing procedures. The major part of PN adult patients can be treated with commercialized products. Therefore, PN treatment is sensibly facilitated. Nevertheless, individual PN compounding remains needed in selected patients, especially in children or neonates with their body growth requirements but also adults with home PN and/or after mesenteric infarction. However, such a service is available only in particular and experienced hospital pharmacies or compounding centers often also challenged for an appropriate logistic [2]. The evidence is still lacking whether an individualized tailor-made PN regime provides better outcome in neonates, acute critically ill patients or for patients in the home setting compared to a standardized commercial AiO PN. The debate concerning the individual energy, macro- and micronutrient requirements is still ongoing [41,42].

## 8. Stability and Compatibility

Stability defines that the admixture components do not degrade in excess (e.g., <10%). Compatibility defines that these components do not physico–chemically interact with each other over a defined time. Stability and compatibility must last from compounding to delivery, up to the declared expiry date, and the administration under defined conditions. Both aspects are critical for the quality, efficacy and safety of the product [43] There are many physicochemical interactions occurring: instabilities (emulsion), solubility (precipitations), photo-induced (catalytic reactions), thermic reactions, material interactions (sorption, permeation), and chemical reactions (oxidation, reduction, hydrolysis, polymerization, decarboxylation, complexation, lipid peroxidation, Maillard reaction, etc.). A decrease in pH below 5.0 seriously compromises lipid emulsion stability. The first visible sign of decreased homogeneity of the oil-in-water emulsion is creaming (appearance of a white upper layer) [21]. Creaming is reversible by gentle mechanical shaking and occurs without a significant change in the mean particle size nor the particle size distribution. Such AiO PN admixture can be safely administered to the patient. The coalescence is the next deterioration step, which is not anymore reversible. Larger lipid droplets increasingly form over time and may be visible at the surface [21]. Coalescence may be measured by a light extinction method or validated microscopic analysis [44,45] Such emulsions should no more be administered to patients as they may induce adverse effects (e.g., inflammation from phagocytized fat globules, or embolism) [46]. The ultimate step of the on-going droplet enlargement is the emulsion breaking or cracking, where large fat globules separate from the dispersed oil-in-water phase [21]. Administration of such formulations is prohibited. These interactions occur between the components present in AiO PN admixture. They can become critical upon admixing and administration of higher amounts of electrolytes leading to precipitates when disrespecting the solubility product, which is influenced by specific conditions in the PN admixture (volume, pH, temperature, chelating components like AA for cations) [2]. Administration of parenterals containing precipitates may cause small vessels occlusion and consecutive organ damages, and may even become fatal when such precipitates are delivered e.g., into the lung [2]. Precipitates may as well occlude central venous catheter [2,16]. Critical concentrations of divalent cations (mainly Mg^2+^ and Ca^2+^) in presence of inorganic phosphate lead to precipitation of insoluble phosphate salts. High requirements of such electrolytes exist in neonates but also in adults are incompatible in the amount needed since compatible concentrations for AiO PN admixture are overreached [2]. Such incompatibilities constitute medication errors and are preventable by careful expert proof and choice of appropriate formulations and dose of an individual AiO admixture by the pharmacist [34]. Replacing inorganic salts by organic ones may sometimes also prevent such insolubilities like organic (not hydrolyzed) glycerophosphates [6]. As a rule of thumb, monovalent (mainly Na^+^ and K^+^) and divalent (mainly Mg^2+^ and Ca^2+^) cations concentrations in AiO admixtures should not exceed 130 mmol/L and 8 mmol/L, respectively (relative ratio of about 15 between mono- and divalent cations) [21,47].

Compatibility and stability of calcium and phosphate in PN admixtures depend on many factors, such as concentrations, pH, nature of the salts (inorganic or organic), presence/concentration of electrolytes, composition and concentration of the AA solution, lipid emulsion, mixing order, temperature, storage, and hanging time since admixing [21]. High concentrations of calcium and inorganic sodium or potassium phosphate (mono- or dibasic) salts show a greater risk of precipitation at room temperature due to the variation in solubility products at different pH (factor 60). The more acidic pH of peripheral PN admixtures (due to lower AA and/or glucose concentrations and increased lipid concentration) compared to central AiO PN admixtures (high osmolarity) also reduces calcium phosphate solubility [21]. Low infusion rates and room temperature, e.g., in PN admixtures for neonates, contribute to increase the risk of precipitation, eventually occurring during administration and consecutively causing severe organ damages, especially in the lungs. Calcium phosphate precipitation may also occur when iron dextran or bicarbonate are added to PN or infused through the same line without sufficient previous line rinsing [21]. Concentrations of organic calcium (e.g., gluconate) should not exceed 2.5 mmol/L in adult formulations with low osmolarity. Phosphate concentration (mono- and dibasic sodium phosphate salts) should not exceed 15 mmol/L [48]. Organic phosphate salts (e.g., glucoso-1-phosphate or glycerol phosphate) should be used in case of increased needs for calcium and phosphate (e.g., children or neonates). However, organic salts are prone to hydrolysis, possibly releasing highly incompatible inorganic phosphate [49].

Another critical reaction partner is the lipid emulsifier lecithin, a negatively charged natural, large molecular AiO PN component, important to control and stabilize the lipid droplet size, and their size distribution in intravenous formulations. Eventually, the stability of the emulsion is most decisive for the overall stability and safety of an individual AiO PN admixture. Polyvalent cations may react and neutralize the negatively charged phosphate moieties of lecithin at the lipid droplet surface (zeta potential) which is intended to hinder (reversible) aggregation of oil droplets (oil droplet agglomerates). Over time, agglomerates can further develop to irreversible oil droplet coalescence. The enlarged oil droplets (>5 μm diameter) are able to obstruct small blood vesicles (lipid embolism) [2]. Breaking apart of the emulsion and oiling out can occur, which has to be avoided strictly, whereas the initial oil droplet formation is potentially visible at the surface of an AiO PN admixture [2]. Their detection is challenging, and microscopic analysis might be a better approach [50]. Even though this microscopic technique is promising, it is not a recommended method to date. The United States pharmacopoeia recommends measuring the volume-weighted percent of fat droplets greater than 5 μm or PFAT5, must not exceed 0.05% of the total fat (PFAT5) [51] This measurement is usually performed by the manufacturer in an accredited laboratory and not in the daily analytical routine of hospital pharmacies [21]. Even small amounts of trace elements (μM concentration) or locally higher concentration upon admixing of components in a wrong order may influence the emulsion stability and safety. Solubilizers for lipophilic drugs used as vehicles in parenteral drug formulations when admixed or in contact with AiO PN may rise the emulsion stability. This negatively affects the lipid clearance in the body of administered TG from the AiO PN admixture since oil-in-water emulsion becomes meta-stable and thus resist to greater extent to enzymatic plasma lipid clearance [4].

### 8.1. Vitamin Stability

Some vitamins (e.g., vitamins A, B1, B2, B6, C and K) are chemically unstable and are easily oxidized with air and light. Vitamin A as a lipophilic compound may also interact with the PN container or the infusion set (absorption and/or adsorption). Vitamin B1 and B6 are unstable in presence of oxygen and in direct interaction with trace elements. Vitamin C is also easily degraded to oxalic acid reacting with calcium to form calcium oxalate precipitate [52]. Clinical deficiencies may occur due to such instabilities. Undesired degradation products (e.g., oxalate) may also be infused. Interactions between labile vitamins and PN components are manifold and therefore difficult to extrapolate in theory. Vitamins may only be added to PN AiO admixtures if specific and sufficient stability data are available. In best practice, vitamins should be added to AiO just before or at the very end of the PN administration [21]. This in order to reduce interaction time and eventual time-dependent degradation. Fat-soluble vitamins (A, D, E, K) can be added to lipid-containing AiO admixtures or into lipid infusions. They may be administered weekly or monthly (vitamin K) according to their storage in the body. Vitamins may be protected from light exposure and degradation by lipid emulsions, however, they are themselves prone to peroxidation. Light protection of AiO admixture containers containing vitamins is thus generally recommended during storage and administration [21]. Adding combined admixture of trace elements and vitamins hides high incompatibility potential (e.g., iron and vitamin C) and is therefore not recommended without specific stability data. Commercial multivitamin preparations can be safely administered as separate infusion or even as a slow bolus injection between daily administration of PN portions [53].

### 8.2. Trace Elements Compatibility

Copper cysteinate, iron phosphate, and formation of insoluble elemental selenium from selenite reduction with vitamin C are the best-known compatibility issues with trace elements [54]. Commercial trace elements solutions show very acidic pH (pH 2), which is critical for lipid stability. Emulsion stability and peroxidation are also negatively influenced by polyvalent cations (e.g., iron, copper, selenium, chrome and zinc) [38,49,55,56]. TG hydrolysis or admixing of trace elements may cause critical pH drop (below pH 5) hampering the emulsion stability. Trace elements should therefore never be added directly to lipid emulsion neither upon compounding nor in a multi-chamber bag. The pH of parenteral products may vary between manufacturers, making it difficult to extrapolate data from one brand to another. AA due to their pH buffering capacity may positively contribute to lipid emulsion stability. AA also have the ability to complex polyvalent cations like electrolytes or trace elements (chelation) avoiding bridging between negatively charged lipid droplets. During compounding or admixing, electrolytes and/or trace elements should therefore be added into the AA solution.

## 9. Artificial Nutrition and Drug Admixture

### 9.1. Drug Administration via Feeding Tube

Whenever drug administration occurs via a feeding tube, basic pharmaceutical questions arise to implement safe and efficient therapy: Can the tablet be crushed, or the capsule opened? How should a therapy plan ultimately be put into practice? Drug delivery via a feeding tube is an interprofessional challenge and requires special expertise from all involved stakeholders. Medical, nursing and pharmaceutical aspects have to be considered. The versatility of orally available drug formulations is large and includes specific forms with modified drug release or protective tablet coatings. Interactions between drugs, nutrients, and the human organism can lead to physicochemical reactions and pharmacological changes affecting treatment’s efficacy and safety [57]. The prevention and handling of such interactions requires pharmaceutical advice and expertise. Incorrect administration of EN and/or medication due to improper handling must be considered as avoidable medication errors. While various international guidelines have been developed for the nutrition of patients, recommendations for the administration of drugs via feeding tubes are primarily based on empirical values [58]. Information on the accessibility of drugs to medications are not provided as standard information and therefore available only by means of extensive literature research. In 2012, Prohaska et al. found that information on the administration of antiretroviral drugs via feeding tube was only reported in 63% of the cases [59]. Significant problems may occur if drugs galenic is changed, e.g., when a tablet is crushed instead of swallowed. Fatalities have for example been reported after a retarded nifedipine preparation was repeatedly unduly crushed, and administered via feeding tube [60]. Enteral tubes are challenging for the caregivers, both during the hospital stay and after discharge. Resulting ambiguity in the care responsibility can endanger patient safety. Nowadays, tubes made of polyurethane or silicone should be used for EN. These materials guarantee a minimal foreign body sensation and good compatibility over a longer lying time. Polyvinyl chloride (PVC) should be used only for short periods because of the risk of pressure necrosis due to the washing out of the plasticizers [61,62]. Interactions between the tube material and the active ingredients of EN are common. PVC may for example retain relevant levels of carbamazepine, clonazepam, diazepam, phenytoin or tacrolimus, leading to treatment failure [63,64]. The position of the tube tip has a decisive influence on the release and absorption of active ingredients (pH stomach = 1–2, pH duodenum/jejunum = 7–8). Acid-sensitive drugs are destroyed in the acidic stomach environment. If a drug with an enteric coating is crushed and administered via a gastric tube, this leads to a loss of effect, since the drug is early degraded by the gastric acid. It is recommended to avoid administering medication through a jejunal or duodenal tube. In the case of jejunal feeding tube placement, the total amount of fluid used in drug administration should thus not exceed 50 mL. A time gap of 30 min should be kept between the individual bolus doses or the next application; otherwise, it can lead to diarrhea. It should further be taken into account that the tube tip does not have to be the site of the active ingredient intake, and that the passage time in the gastrointestinal tract differs significantly from the oral intake. Consequently, administration of drugs via feeding tube may result in a lower effectiveness or higher risk of side effects compared to the oral route. Blood/urine levels of drugs with narrow therapeutic range administered via a feeding tube should be regularly checked.

#### 9.1.1. Administration of Drugs via Feeding Tube

**Figure 2:**
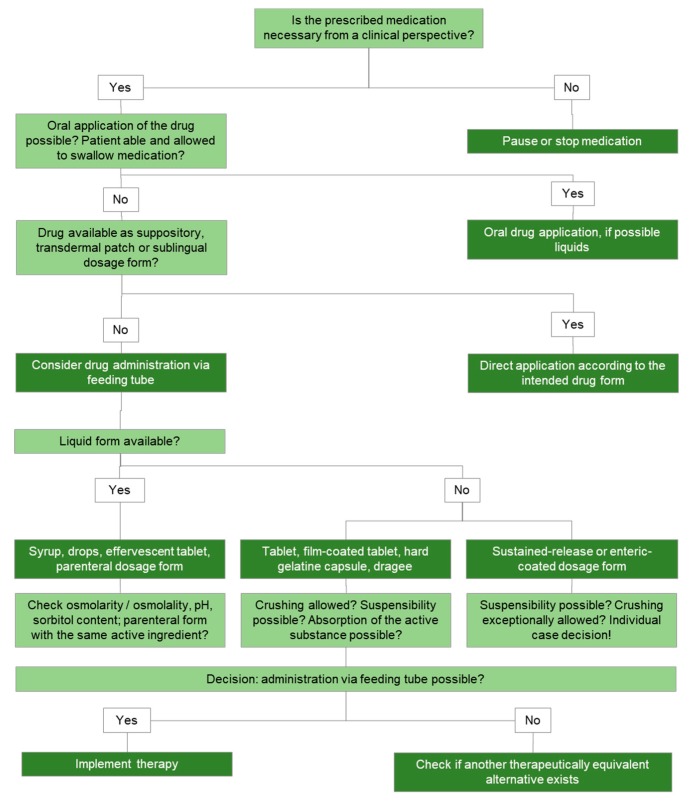
Algorithm for the evaluation of drug administration via feeding tube.

From a methodical point of view, drug administration via feeding tube is challenging and offers the possibility of interprofessional medication review [65]. This is ideally be done before the final medication prescription, ensuring a safe and efficient administration. In the course of a visit on the ward or in the nursing home, the pharmacist clarify with the physician whether the patient really needs all prescribed drugs in his current situation. Any deprescribing relieves the patient and nursing staff, and reduces complications. Decision for the administration of drug via feeding tube is supported in a structured way as shown in Figure 2. The oral route should be preferred for the drug administration. The patient’s ability to swallow has to be evaluated, and regularly promoted, as it may also depend on the form of the day. Depending on the patient’s existing ability to swallow, solid dosage forms are replaced by sublingual, liquid, mouth-melting, or transdermal forms. If the patient can swallow liquids, medications should be offered in liquid form or as dispersible tablets (physiological route and less complication potential than via tube). Medicines in liquid dosage form are moreover uncomplicated to give over the tube than solid forms. Numerous interactions and reactions involving drug products, nutrients, and tube devices exist despite the use of solid drug formulation (lower reactivity compared to a dissolved drug delivery system).

Rectal, transdermal, nasal, sublingual or parenteral drug administration should be considered as alternatives to drug application via feeding tube [66]. If no alternative medication is available, an individualized formulation from the pharmacy may be considered. The tube system itself is equally crucial to further assessment. A PEG is more suitable for drug administration than a transnasal or a fine needle catheterized jejunostomy tube because of its brevity and width. The diameter and the location of the tip of the tube are just as important. The osmolality of enteral nutrition is on average 300 mosmol/kg and must not exceed 500–600 mosmol/kg. The stomach tolerates osmolality of up to 1000 mosmol/kg. Liquids with higher osmolality lead to diarrhea (due to a high volume of fluid and electrolyte that cannot be absorbed in the small intestine), nausea, flatulence (due to delayed gastric emptying), or vomiting. To prevent osmotic diarrhea or vomiting, drug formulations have to be diluted with water to isotonic or slightly hypertonic (<400–500 mosmol/kg) solutions to be given through the feeding tube. Even stricter isotonicity is required in case of small intestine tubes, due to the lack of dilution processes in the mouth, esophagus and stomach. Since the intestine, in contrast to the stomach has no storage function, no liquid amounts of more than 50 mL should be given as a bolus directly into the intestine. This significantly restricts the administration of several drugs with according tube position.

#### 9.1.2. Safety Issues in the Administration of Drugs via Tube

The preparation and administration of carcinogenic, mutagenic drugs or drugs toxic to reproduction, so-called CMR drugs require special attention. Thus, the respective hazard risk handling with cytostatics, anti-infectives/virustatics, hormones, and immunosuppressants must be individually assessed. Work must then be carried out by trained staff, with the necessary protective measures (e.g., gloves, dust mask, protective googles). Patients’ relatives and caregivers must be made aware of the relative risk potential. Pregnant or breastfeeding women have to be relieved of such activities. In addition, patients, relatives and caregivers must be informed about safer alternatives dosage forms as liquid dosage forms for example carry less risk than tablets that have to be divided or crushed. Open handling of CMR substances should always be avoided. Pharmacies having dedicated facilities may provide CMR drugs as capsules or pre-filled syringes to minimize drug exposure. The increasing number of oral cancer therapies on the drug market will lead to increased handling questions in the outpatient care.

### 9.2. Drug Admixture to PN

Extrapolation of AiO PN admixtures stability data from the literature is rarely possible because of the high number and concentration variabilities of components in an individual PN nutrition regimen (Table 3). This is even more complicated in case of drug admixing. The multitude and the concentration dependence of interactions create a multifactorial system not to be assessed by theoretical calculations only. Generation of pharmaceutical stability data may therefore be required for specific cases for documentation and reliability purposes. Sensitive and validated laboratory methods are available in tertiary care hospitals, which may be useful for components of an AiO PN admixtures and concomitant therapy but have to be adapted for the specific in vitro investigation. Such evaluation tools may help to assess the possibility to admix a specific item into a defined AiO PN composition or to check an individual nutrient and/or drug interaction upon request from the clinic in a reasonable and relative short time period [2]. Light microscopy may for example be applied to assess oil-in-water (oil droplet sizing) emulsions. Even though the admixing of drugs to AiO PN admixtures is not recommended, it may be needed in some cases. Compatibility and stability assessment of such combinations may be performed by means of defined drug analytic methods for quantification, adapted from blood plasma or serum to PN matrix [6,50]. Simple test methods have to be elaborated and validated to be used for stability and compatibility assessment of individual admixes to AiO PN regimens upon request [2,16,50].

The following points should be considered as good practice using concomitant parenteral drug therapy in PN patients [57,67]:

- Check if the medication is really needed.
- Ask for pharmaceutical advice, ideally from the nutrition support team when therapy regimen are complicated or when drug admixing to PN admixture is considered.
- Admix compatible drugs to PN only just before administration in order to minimize interactions.
- Document procedure, creating a database to control and reference drug-PN admixing interventions.
- If possible collect samples for later analysis and evaluation.
- Use alternative infusion lines (inclusive of other catheter lumen) for drug administration whenever possible. In absence of a separate line, intermittent intravenous drug administration in saline or glucose solutions may be considered. Sufficient catheter rinsing before and after drug administration is mandatory. Special attention is needed for metabolic adverse effects when stopping PN administration because of intermittent drug administration. Insulin stimulation induced by glucose infusion may for example be reduced by lowering the PN administration rates over the last half hour before stopping.

## 10. Monitoring of Artificial Nutrition

Regular, defined and appropriate assessment may help to avoid respectively decrease the metabolic complications of artificial nutrition. This should include the determination of (specific) individual nutrients, the monitoring and the course of the underlying disease and laboratory testing in addition to the routine clinical assessment. Specific laboratory tests of artificial nutrition-associated parameters include ([5,6,7]: Hematological testing, lipid status, liver and kidney function (especially in PN), glucose, sodium, potassium, calcium, magnesium, phosphate, CRP, and iron status. Monitoring of anthropometrics such as body weight, body mass index (BMI) and hydration status should as well be performed to ensure the metabolic tolerance of artificial nutrition. Extended and frequent monitoring is required at the initiation of PN for up to two months. Monitoring of long-term, clinically stable PN patients is recommended every 3 to 6 months [6].

## 11. Home Artificial Nutrition

Some patients require a long-term artificial nutrition, if possible performed in a home setting when no other hospital treatment is needed. The convenient and mostly nocturnal administration enables patients to have a nearly “normal” everyday life and in many cases to keep on working. Patients receiving home artificial nutrition show enhanced quality of life and better social integration [68]. Home artificial nutrition complications rates are lower than in hospital setting, possibly because of the highly trained aseptic handling skills and to the more stable health patients’ situation [7]. Nevertheless, it should undergo regular supervision by NSTs and a reliable reporting on critical incidences or health problems has to be in place. European data show that home EN incidence is around 400 per million and home PN incidence around 5 per million people per year. These differences are most likely due to different healthcare systems and resources [3,5,68,69]. To draw conclusions how to better care these home artificial nutrition patients, national registries are necessary to improve and to benchmark the different national experiences [2].

## 12. Conclusions

The provision of artificial nutrition is a necessary when oral/enteral feeding is insufficient or impossible. With the development of artificial nutrition, administration of EN and mainly PN became convenient and safer, even possible in a home setting. Well-controlled procedures and steady monitoring of the patients by an experienced NST are key issues in the successful management of artificial nutrition, from the prescription (medical care) to through the validation (pharmaceutical care) to the implementation (nursing care) of the therapy. Proper handling, compounding and concomitant drug administration are of the highest relevance for therapeutic success and patient’s safety, in EN as well as in PN therapy.

## Figures and Tables

**Table 1:** Epidemiologic data from 3239 patients with chronic intestinal failure in countries around the world, from Pironi et al. [1].

Type of Chronic Intestinal Failure	Underlying Disease
Benign chronic intestinal failure (*n* = 2919, 90.1%)	Crohn’s disease (22.4%) Mesenteric ischemia (17.7%) Surgical complications (15.8%) Primary chronic intestinal pseudo-obstruction (9.7%) Post-radiation enteritis (7.3%) Others (21.3%, with <3% each-one) Not reported (5.9%)
Malignant chronic intestinal failure (*n* = 320, 9.9%)	Type of active cancer not specified (62%) Gastrointestinal cancer (28%) Extra-abdominal cancer (10%) Concurrent enteritis due to radio- or chemotherapy (5%) Peritoneal carcinomatosis (12%)

**Table 2:** Catheter- and parenteral nutrition (PN)-related complications [2].

Type	Rates Measures Per Catheter Year (95% Confidence Interval)
Catheter sepsis	0.34 (0.32–0.37)
Catheter occlusion	0.07 (0.06–0.08)
Central vein thrombosis	0.03 (0.02–0.03)
Liver/biliary issues	
Mild	0.42 (0.27–0.63)
Severe	0.02 (0.01–0.06)
Metabolic bone disease	0.05 (0.01–0.15)

**Table 3:** Advices to the possible drug admixture to AiO PN admixture.

Advice Not to Admix	lipophilic drugs with solubilizes such as cremophors, tweens, etc. drugs with rapid chemical or physical instabilities drugs with narrow therapeutic indices (cytotoxics, etc.) macromolecular recombinant biotech drugs, (signaling proteins, monoclonal antibodies or fusion proteins) synthetic non-biological complexes (nanomedicines) drugs with short elimination half-lives and dosage intervals not adjusted to the PN administration period
Admixing May be Possible	stability documentation is sufficiently available no ingredients with potential incompatibilities (CAVE polyvalent cations) large therapeutic index drugs (analgesics, sedatives, H_2_-antagonists, etc.) simple stability testing is possible (pH, emulsion characterization and visual inspection) well-established, often used, and physico–chemically fully characterized small-molecular drugs
